# Two Remarks on Graph Norms

**DOI:** 10.1007/s00454-021-00280-w

**Published:** 2021-02-16

**Authors:** Frederik Garbe, Jan Hladký, Joonkyung Lee

**Affiliations:** 1 Institute of Mathematics of the Czech Academy of Sciences, Žitná 25, 115 67 Prague, Czechia; 2 Department of Mathematics, University College London, Gower Street, London, WC1E 6BT UK

**Keywords:** Graph norms, Graph limits, Graphons

## Abstract

For a graph *H*, its homomorphism density in graphs naturally extends to the space of two-variable symmetric functions *W* in $L^p$, $p\ge e(H)$, denoted by *t*(*H*, *W*). One may then define corresponding functionals $\Vert W\Vert _{H}\,{:}{=}\,|t(H,W)|^{1/e(H)}$ and $\Vert W\Vert _{r(H)}\,{:}{=}\,t(H,|W|)^{1/e(H)}$, and say that *H* is (semi-)norming if $\Vert \,{\cdot }\,\Vert _{H}$ is a (semi-)norm and that *H* is weakly norming if $\Vert \,{\cdot }\,\Vert _{r(H)}$ is a norm. We obtain two results that contribute to the theory of (weakly) norming graphs. Firstly, answering a question of Hatami, who estimated the modulus of convexity and smoothness of $\Vert \,{\cdot }\,\Vert _{H}$, we prove that $\Vert \,{\cdot }\,\Vert _{r(H)}$ is neither uniformly convex nor uniformly smooth, provided that *H* is weakly norming. Secondly, we prove that every graph *H* without isolated vertices is (weakly) norming if and only if each component is an isomorphic copy of a (weakly) norming graph. This strong factorisation result allows us to assume connectivity of *H* when studying graph norms. In particular, we correct a negligence in the original statement of the aforementioned theorem by Hatami.

## Introduction

One of the cornerstones of the theory of quasirandomness, due to Chung et al. [1] and to Thomason [10], is that a graph is quasirandom if and only if it admits a random-like count for any even cycle. A modern interpretation of this phenomenon is that the even cycle counts are essentially equivalent to the Schatten–von Neumann norms on the space of two variable symmetric functions, which are the natural limit object of large dense graphs. Indeed, Lovász [7] asked the natural question whether other graph counts can also induce a similar norm, which motivated Hatami’s pioneering work [5] in the area. Since then, graph norms have been an important concept in the theory of graph limits and received considerable attention. For instance, Conlon and the third author [2] obtained a large class of graph norms, Král’ et al. [6] proved that edge-transitive non-norming graphs exist, and very recently, the first author with Doležal et al. [4] linked graph norms to the so-called step Sidorenko property.

The current note contributes further to this emerging theory of graph norms. We recall the basic definitions given in Hatami’s work [5] with slight modifications taken from [8]. Let $\Omega$ be an arbitrary standard Borel space with an atomless probability measure $\nu$. Whenever we consider a subset of $\Omega$, we tacitly assume that it is measurable. We denote by ${\mathscr {W}}$ the linear space of all bounded symmetric measurable functions $W:\Omega ^2\rightarrow {\mathbb {R}}$. Also let ${\mathscr {W}}_{\ge 0}\subseteq {\mathscr {W}}$ be the set of non-negative functions in ${\mathscr {W}}$. Recall that functions in ${\mathscr {W}}_{\ge 0}$ that are bounded above by 1 are called *graphons*, and arise as limits of graph sequences [9]. Let *H* be a graph on the vertex set $\{v_1,\ldots ,v_n\}$. Given a symmetric measurable real-valued function *W* on $\Omega ^2$, set

1.1 $$\begin{aligned} t(H,W)\,{:}{=}\,\int \limits _{x_{1}\in \Omega }\!\!\ldots \!\!\int \limits _ {x_{n}\in \Omega }\,\prod _{\{v_{i},v_{j}\}\in E(H)}\!\!\!W(x_{i},x_{j})\,d\nu ^{\otimes n}. \end{aligned}$$

Let ${\mathscr {W}}_H$ (resp. ${\mathscr {W}}_{r(H)}$) be the set of those symmetric measurable functions $W:\Omega ^2\rightarrow {\mathbb {R}}$ for which *t*(*H*, *W*) (resp. *t*(*H*, |*W*|)) is defined and finite. Obviously, ${\mathscr {W}}_H$ is a subspace of ${\mathscr {W}}_{r(H)}$, and Hölder’s inequality immediately proves that $L^p(\Omega ^2)$ is contained in ${\mathscr {W}}_{r(H)}$ whenever $p\ge e(H)$.

We then say that *H* is (*semi-*)*norming* if $\Vert \,{\cdot }\,\Vert _H\,{:}{=}\,|t(H,\,{\cdot }\,)|^{1/e(H)}$ is a (semi-)norm on ${\mathscr {W}}_H$. Likewise, we say that *H* is *weakly norming* if $\Vert \,{\cdot }\,\Vert _{r(H)}\,{:}{=}\,t(H,|\,{\cdot }\,|)^{1/e(H)}$ is a norm on ${\mathscr {W}}_{r(H)}$. Since ${\mathscr {W}}$ is a dense subset of the Banach space^1^$({\mathscr {W}}_H,\Vert \,{\cdot }\,\Vert _H)$, this definition does not depend on whether we work in the Banach space $({\mathscr {W}},\Vert \,{\cdot }\,\Vert _H)$ or $({\mathscr {W}}_H,\Vert \,{\cdot }\,\Vert _H)$. Analogously, in the definition of weakly norming property, ${\mathscr {W}}_{r(H)}$ can be replaced by ${\mathscr {W}}$. Note that, as the names suggest, norming graphs are semi-norming and semi-norming graphs are weakly norming.

Prominent examples of norming graphs are even cycles $C_{2k}$ and complete bipartite graphs $K_{2n,2m}$ with an even number of vertices per partite set. Seminorming graphs that are not norming are stars with an even number of edges. Examples of weakly norming graphs that are not (semi-)norming are complete bipartite graphs $K_{n,m}$ with $m>1$ being odd. We refer the reader to [2, 5, 8] for more details and examples.

In what follows, we shall give short proofs of two results concerning (weakly) norming graphs. Firstly, we study basic geometric properties of the space $({\mathscr {W}}_{r(H)},\Vert \,{\cdot }\,\Vert _{r(H)})$. The definitions of uniform smoothness and uniform convexity will be precisely given in the next section.

### Theorem 1.1

Let *H* be a weakly norming graph. Then the normed space $({\mathscr {W}}_{r(H)},\Vert \,{\cdot }\,\Vert _{r(H)})$ is neither uniformly smooth nor uniformly convex.

This answers a question of Hatami, who proved that $({\mathscr {W}},\Vert \cdot \Vert _{H})$ *is* uniformly smooth and uniformly convex whenever *H* is semi-norming and asked for a counterpart of his theorem for weakly norming graphs.

Theorem 1.1 not only answers a natural question arising from a functional-analytic perspective, but is also meaningful in the theory of quasirandomness. In [4], Hatami’s theorem about uniform convexity and smoothness (see Theorem 2.2 for a precise statement) is the key ingredient in proving that every norming graph has the ‘step forcing property’. By inspecting the proof in [4], one may see that the same conclusion for weakly norming graphs *H* (except forests) could also be obtained if $\Vert \,{\cdot }\,\Vert _{r(H)}$ defined a uniformly convex space. However, Theorem 1.1 proves that such a modification is impossible.

Secondly, we prove a strong ‘factorisation’ result for disconnected weakly norming graphs.

### Theorem 1.2

A graph *H* is weakly norming if and only if all its non-singleton connected components are isomorphic and weakly norming. The same statement with weakly norming replaced by either semi-norming or norming also holds.

The ‘if’ direction is obvious, since $|t(H,W)|^{1/e(H)}=|t(H',W)|^{1/e(H')}$ whenever $W\in {\mathscr {W}}$ and *H* is a vertex-disjoint union of copies of $H'$ and an arbitrary number of isolated vertices, but the converse is non-trivial. Theorem 1.2 corrects a negligence that assumes connectivity of graphs without stating it, which in fact appeared in Hatami’s work [5] and Lovász’s book [8] which study graph norms. We also remark that for Sidorenko’s conjecture, a major open problem in extremal combinatorics, even a weak factorisation result—such as each component of a graph satisfying the conjecture also satisfies it—is unknown, even though weakly norming graphs satisfy the conjecture. In fact, Conlon and the third author [3, Corr. 1.3] proved that the weak factorisation result, if it exists, implies the full conjecture.

## Moduli of Convexity and Smoothness

We begin by recalling the definitions of moduli of convexity and moduli of smoothness of a normed space.

### Definition 2.1

Let $(X,\Vert \,{\cdot }\,\Vert )$ be a normed space. The *modulus of convexity of* *X* is a function ${\mathfrak {d}}_{X}:(0,2]\rightarrow {\mathbb {R}}$ defined by

2.1 $$\begin{aligned} {\mathfrak {d}}_{X}(\varepsilon )&\,{:}{=}\,\inf {\biggl \{ 1- \biggl \Vert \frac{x+y}{2}\biggr \Vert :x,y\in X,\,\Vert x-y\Vert \ge \varepsilon , \,\Vert x\Vert =\Vert y\Vert =1\biggr \}} . \end{aligned}$$

The *modulus of smoothness of* *X* is a function ${\mathfrak {s}}_{X}:(0,\infty )\rightarrow {\mathbb {R}}$ defined by

2.2 $$\begin{aligned} {\mathfrak {s}}_{X}(\varepsilon )&\,{:}{=}\,\sup {\biggl \{ \frac{\Vert x+y\Vert +\Vert x-y\Vert -2}{2}:x,y\in X,\,\Vert x\Vert =1,\,\Vert y\Vert =\varepsilon \biggr \}} . \end{aligned}$$

The normed space $(X,\Vert \,{\cdot }\,\Vert )$ is *uniformly convex* if ${\mathfrak {d}}_{X}(\varepsilon )>0$ for each $\varepsilon >0$ and is *uniformly smooth* if ${\mathfrak {s}}_{X}(\varepsilon )/{\varepsilon }\rightarrow 0$ as $\varepsilon \searrow 0$. For convenience, we write ${\mathfrak {d}}_{H}$, ${\mathfrak {s}}_{H}$, ${\mathfrak {d}}_{r(H)}$, and ${\mathfrak {s}}_{r(H)}$ instead of ${\mathfrak {d}}_{{\mathscr {W}}_H}$, ${\mathfrak {s}}_{{\mathscr {W}}_H}$, ${\mathfrak {d}}_{{\mathscr {W}}_{r(H)}}$, and ${\mathfrak {s}}_{{\mathscr {W}}_{r(H)}}$, respectively.

Hatami [5] determined ${\mathfrak {d}}_{H}$ and ${\mathfrak {s}}_{H}$ for connected norming graphs *H* up to a multiplicative constant by relating them to the moduli of convexity and of smoothness of $\ell ^p$-spaces, which are well understood.

### Theorem 2.2

([5, Thm. 2.16]) For each $m\in {\mathbb {N}}$, there exist constants $C_m,C'_m>0$ such that the following holds: let *H* be a connected semi-norming graph with *m* edges. Then the Banach space $({\mathscr {W}}_H,\Vert \,{\cdot }\,\Vert _H)$ satisfies $C_m\cdot {\mathfrak {d}}_{\ell ^m}\le {\mathfrak {d}}_{H}\le {\mathfrak {d}}_{\ell ^m}$ and ${\mathfrak {s}}_{\ell ^m}\le {\mathfrak {s}}_{H}\le C'_m\cdot {\mathfrak {s}}_{\ell ^m}$.

Since for each $p\in (1,+\infty )$ it is well known that the $\ell ^p$-space is uniformly convex and uniformly smooth, one obtains the following.

### Corollary 2.3

Let *H* be a connected semi-norming graph. Then the Banach space $({\mathscr {W}}_H,\Vert \,{\cdot }\,\Vert _H)$ is uniformly convex and uniformly smooth.

The connectivity of *H* in Theorem 2.2 was in fact neglected in the original statement in [5], but it is certainly necessary. For example, by taking a disjoint union of two isomorphic norming graphs with *m*/2 edges (assume *m* is even), one obtains another norming graph with *m* edges that gives exactly the same norm, whose correct parameters in Theorem 2.2 are ${\mathfrak {d}}_H=\Theta ({\mathfrak {d}}_{\ell ^{m/2}})$ and ${\mathfrak {s}}_H =\Theta ({\mathfrak {s}}_{\ell ^{m/2}})$. Indeed, in Theorem 4.1 below we obtain a general statement without assuming connectivity, by using Theorem 1.2. But first, let us point out the negligence in [5] which causes that the proof of Theorem 2.2 does not work for disconnected graphs. This subtle error lies in proving ${\mathfrak {d}}_{H}\le {\mathfrak {d}}_{\ell ^m}$ and ${\mathfrak {s}}_{\ell ^m}\le {\mathfrak {s}}_{H}$ by claiming that the Banach space $({\mathscr {W}}_H,\Vert \cdot \Vert _H)$ contains a subspace isomorphic to $(\ell ^m,\Vert \cdot \Vert _m)$. Here we give a full proof of the claim, which in turn reveals where the connectivity of *H* is used. To this end, we introduce the following notation, which will also be useful in Sect. 3.

### Definition 2.4

Let $\Omega$ be partitioned as $\Omega =\Omega _1\sqcup \Omega _2\sqcup \ldots$ with countably many parts such that $\nu (\Omega _i)=2^{-i}$ for every $i\in {\mathbb {N}}$. For each $m\in {\mathbb {N}}$, $\gamma >0$, ${\mathbf {a}}=(a_1,a_2,\ldots )\in \ell ^m$, $W_{\gamma ,{\mathbf {a}}}$ denotes the function satisfying $W_{\gamma ,{\mathbf {a}}}(x,y)=2^{i\gamma }a_i$ whenever $(x,y)\in \Omega _i^2$ and $W_{\gamma ,{\mathbf {a}}}=0$ outside $\bigcup _i\Omega _i^2$.

Suppose that *H* is a norming graph with *n* vertices and *m* edges. In particular this implies that *m* is even (see [8, Exer. 14.8]). The map ${\mathbf {a}}\mapsto W_{{n}/{m},{\mathbf {a}}}$ is linear, and thus, proving that this map preserves the respective norms is enough to conclude that the subspace spanned by $W_{{n}/{m},{\mathbf {a}}}$ is isomorphic to $\ell ^m$. For each ${\mathbf {a}}=(a_1,a_2,\ldots )\in \ell ^m$,

$$\begin{aligned} \Vert {\mathbf {a}}\Vert ^m_m=\sum _i a_i^m=\sum _i \frac{(2^{in/m}a_i)^m}{2^{in}}=t(H,W_{{n}/{m},{\mathbf {a}}}). \end{aligned}$$

Indeed, if $x_1,\ldots ,x_n$ do not fall into any single $\Omega _i$, connectedness of *H* implies that the product in (1.1) evaluates to 0. Otherwise, if $(x_1,\ldots ,x_n)\in \Omega _i^n$ for some $i\in {\mathbb {N}}$, then $\nu ^{\otimes n}(\Omega _i^n)=2^{-in}$ and the product in (1.1) evaluates to constant $(2^{in/m}a_i)^m$, which proves the last equality. This is exactly where the proof of the claim relies on *H* being connected.

Now, turning to weakly norming graphs, Theorem 1.1 is a direct consequence of the following result.

### Theorem 2.5

Let *H* be a weakly norming graph. Then for each $\varepsilon \in (0,1)$,

- $${\mathfrak {d}}_{r(H)}(\varepsilon )=0$$, and
- $${\mathfrak {s}}_{r(H)}(\varepsilon )\ge \varepsilon /2$$.

For the proof, we introduce a random graphon model that generalises graphon representations of the Erdős–Rényi random graph. Let ${\mathscr {D}}$ be a probability distribution on [0, 1] and let $\Omega =\Omega _1\sqcup \ldots \sqcup \Omega _n$ be an arbitrary partition of $\Omega$ into sets of measure 1/*n*. Denote by ${\mathbb {U}}(n,{\mathscr {D}})$ the random graphon obtained by assigning a constant value generated independently at random by the distribution ${\mathscr {D}}$ on each $(\Omega _i\times \Omega _j) \cup (\Omega _j\times \Omega _i)$, $1\le i\le j\le n$. Although ${\mathbb {U}}(n,{\mathscr {D}})$ depends on the partition $\Omega _1\sqcup \ldots \sqcup \Omega _n$, we shall suppress the dependency parameter as different ${\mathbb {U}}(n,{\mathscr {D}})$’s are ‘isomorphic’ in the sense that there exists a measure-preserving bijection that maps one partition to the other. We use the term *asymptotically almost surely*, or *a.a.s.* for short, in the standard way, i.e., a property ${\mathscr {P}}$ of ${\mathbb {U}}(n,{\mathscr {D}})$ holds a.a.s. if the probability that ${\mathscr {P}}$ occurs tends to 1 as $n\rightarrow \infty$. We write $a=b\pm \epsilon$ if and only if $a\in [b-\epsilon ,b+\epsilon ]$.

### Proposition 2.6

Let ${\mathscr {D}}$ be a probability distribution on [0, 1] and let $d={\mathbb {E}}[{\mathscr {D}}]$. Then for any fixed graph *H*, $U\sim {\mathbb {U}}(n,{\mathscr {D}})$ satisfies $t(H,U)=d^{e(H)}\pm o_n(1)$ a.a.s.

We omit the proof, as it is a straightforward application of the standard concentration inequalities to subgraph densities in Erdős–Rényi random graphs (see, for example, [8, Corr. 10.4]).

### Proof of Theorem 2.5

Throughout the proof, we briefly write $\Vert \cdot \Vert _{r(H)}=\Vert \,{\cdot }\,\Vert$. For $x\in [0,1]$, denote by ${\mathbf {1}}\{x\}$ the Dirac measure on *x*. Set

$$\begin{aligned} {\mathscr {D}}_1\,{:}{=}\,\frac{1}{2} \cdot {\mathbf {1}}\{0\}+\frac{1}{2} \cdot {\mathbf {1}}\{1\}. \end{aligned}$$

Let $U_1$ and $U_2$ be two independent copies of ${\mathbb {U}}(n,{\mathscr {D}}_1)$. Proposition 2.6 then implies a.a.s.

2.3 $$\begin{aligned} \Vert U_{i}\Vert =t(H,U_i)^{1/e(H)}=\frac{1}{2}\pm o_{n}(1), \quad \ \text {for i=1,2.} \end{aligned}$$

For each $i=1,2$, let $U^*_i\,{:}{=}\,U_i/(2\,\Vert U_{i}\Vert )$ be the normalisation of $U_i$ which satisfies $\Vert U^*_{i}\Vert ={1}/{2}$. Then by substituting $U_i=2\,\Vert U_i\Vert \cdot U^*_i$ and using (2.3) we get that

2.4 $$\begin{aligned} \Vert U^*_i-U_i\Vert =|1-2\,\Vert U_i\Vert |\cdot \Vert U^*_i\Vert =o_n(1). \end{aligned}$$

Since the random graphon $|U_{1}-U_{2}|$ is also distributed like ${\mathbb {U}}(n,{\mathscr {D}}_1)$, we again have $\Vert U_{1}-U_{2}\Vert ={1}/{2}\pm o_{n}(1)$ a.a.s. Thus, by the triangle inequality and (2.4), $2U^*_{1}$ and $2U^*_{2}$ are two symmetric functions with $\Vert 2U^*_{1}\Vert =\Vert 2U^*_2\Vert =1$ whose linear combination is always close to the corresponding one of $U_1$ and $U_2$, i.e., for any fixed $\alpha ,\beta \in {\mathbb {R}}$,

2.5 $$\begin{aligned} \bigl |\Vert \alpha U_1+\beta U_2 \Vert -\Vert \alpha U^*_1+\beta U^*_2\Vert \bigr |\le |\alpha |\cdot \Vert U_1-U^*_1\Vert +|\beta |\cdot \Vert U_2-U^*_2\Vert = o_n(1). \end{aligned}$$

In particular, $\alpha =2$ and $\beta =-2$ give $\Vert 2U^*_{1}-2U^*_{2}\Vert \ge \Vert 2U_{1}-2U_{2}\Vert -o_n(1)=1\pm o_{n}(1)$. That is, $2U_1^*$ and $2U_2^*$ are points on the unit sphere that are ‘far’ apart. Setting $\alpha =\beta =1$ in (2.5) gives $|\Vert U_1+U_2\Vert -\Vert U^*_1+U^*_2\Vert |= o_n(1)$, and therefore, for any $0<\varepsilon <1$,

2.6 $$\begin{aligned} {\mathfrak {d}}_{r(H)}(\varepsilon )\le 1-\biggl \Vert \frac{2U^*_1+2U^*_2}{2}\biggr \Vert =1-\biggl \Vert \frac{2U_1+2U_2}{2}\biggr \Vert \pm o_n(1). \end{aligned}$$

Now let

$$\begin{aligned} {\mathscr {D}}_2\,{:}{=}\,\frac{1}{4} \cdot {\mathbf {1}}\{0\}+\frac{1}{2} \cdot {\mathbf {1}}\biggl \{\frac{1}{2}\biggr \}+\frac{1}{4} \cdot {\mathbf {1}}\{1\}. \end{aligned}$$

Then, since $(U_{1}+U_{2})/2$ has distribution ${\mathbb {U}}(n,{\mathscr {D}}_2)$ and ${\mathbb {E}}[{\mathscr {D}}_2]=1/2$, we have by Proposition 2.6 a.a.s. $\Vert U_{1}+U_{2}\Vert =1\pm o_{n}(1)$. Substituting this into (2.6) proves that the modulus of convexity of $\Vert \,{\cdot }\,\Vert$ is 0 for each $\varepsilon \in (0,1)$. For $\varepsilon \in (0,1)$ given in (b), let

$$\begin{aligned} {\mathscr {D}}_3&\,{:}{=}\,\frac{1}{4} ({\mathbf {1}}\{0\}+{\mathbf {1}}\{\varepsilon \}+{\mathbf {1}}\{1-\varepsilon \}+{\mathbf {1}}\{1\})\quad \text { and }\\ {\mathscr {D}}_4&\,{:}{=}\,\frac{1}{4}\biggl ({\mathbf {1}}\{0\}+{\mathbf {1}}\biggl \{\frac{\varepsilon }{2}\biggr \}+{\mathbf {1}}\biggl \{\frac{1}{2}\biggr \}+ {\mathbf {1}}\biggl \{\frac{1+\varepsilon }{2}\biggr \}\biggr ). \end{aligned}$$

The distributions of $|U_{1}-\varepsilon U_{2}|$ and $|U_{1}+\varepsilon U_{2}|/2$ are ${\mathbb {U}}(n,{\mathscr {D}}_3)$ and ${\mathbb {U}}(n,{\mathscr {D}}_4)$, respectively. As ${\mathbb {E}}[{\mathscr {D}}_3]=1/2$ and ${\mathbb {E}}[{\mathscr {D}}_4]=({1+\varepsilon })/4$, Proposition 2.6 yields that, a.a.s., $\Vert 2U_{1}-2\varepsilon U_{2}\Vert =1\pm o_n(1)$ and $\Vert 2U_{1}+2\varepsilon U_{2}\Vert =1+\varepsilon \pm o_n(1)$. Therefore, by (2.5), $\Vert 2U^*_{1}-2\varepsilon U^*_{2}\Vert =1\pm o_n(1)$ and $\Vert 2U^*_{1}+2\varepsilon U^*_{2}\Vert =1+\varepsilon \pm o_n(1)$ a.a.s. Hence, substituting $2U^*_{1}$ and $2\varepsilon U^*_{2}$ into (2.2) gives

$${\mathfrak {s}}_{X}(\varepsilon )\ge \frac{\Vert 2U^*_{1}+2\varepsilon U^*_{2}\Vert +\Vert 2U^*_{1}-2\varepsilon U^*_{2}\Vert -2}{2}=\frac{\varepsilon }{2}\pm o_n(1),$$

which proves (b). $$\square $$

## Disconnected (Semi-)Norming and Weakly Norming Graphs

To be precise, we expand Theorem 1.2 to two parallel statements, also omitting any isolated vertices from *H* (this operation does not change $t(H,\cdot )^{1/e(H)}$).

### Theorem 3.1

(restated) For a graph *H* without isolated vertices, the following holds:

- A graph *H* is weakly norming if and only if all connected components of *H* are isomorphic and weakly norming.
- A graph *H* is (semi-)norming if and only if all connected components of *H* are isomorphic and (semi-)norming.

To prove this theorem, we need some basic facts about weakly norming graphs. Given a graph *H* and a collection ${\mathbf {w}}=(W_e)_{e\in E(H)}\in {\mathscr {W}}^{E(H)}$, define the ${\mathbf {w}}$-*decorated homomorphism density* by

$$\begin{aligned} t(H,{\mathbf {w}})\,\,{:}{=}\,\int \limits _{x_{1}\in \Omega }\!\!\ldots \!\!\int \limits _{x_{n}\in \Omega }\,\prod _{e=ij\in E(H)}\!\!\!W_{e}(x_{i},x_{j}). \end{aligned}$$

That is, we assign a possibly different $W_e$ to each $e\in E(H)$ and count such ‘multicoloured’ copies of *H*. In particular, if $W_e=W$ for all $e\in E(H)$, we obtain $t(H,{\mathbf {w}})=t(H,W)$. Hatami [5] observed that the (weakly) norming property is equivalent to a Hölder-type inequality for the decorated homomorphism density.

### Lemma 3.2

([5, Thm. 2.8]) Let *H* be a graph. Then:

- *H* is weakly norming if and only if, for every $w\in {\mathscr {W}}_{\ge 0}^{E(H)}$,
  $$t(H,{\mathbf {w}})^{e(H)}\le \prod _{e\in E(H)}\!t(H,W_e).$$
- *H* is semi-norming if and only if, for every $w\in {\mathscr {W}}^{E(H)}$,
  $$t(H,{\mathbf {w}})^{e(H)}\le \prod _{e\in E(H)} \!|t(H,W_e)|.$$

As the second inequality is more general than the first one, it immediately follows that every semi-norming graph is weakly norming. Another easy consequence of this characterisation is that, for a weakly norming graph *H*, its subgraph *F*, and $W\in {\mathscr {W}}_{\ge 0}$, we have the inequality

3.1 $$\begin{aligned} t(F,W)\le t(H,W)^{e(F)/e(H)}. \end{aligned}$$

Indeed, one can easily prove this by setting $W_e=W$ for $e\in E(F)$ and $W_e\equiv 1$ otherwise. For yet another application, we use Lemma 3.2 to prove that a weakly norming graph essentially has no subgraph with larger average degree.

### Lemma 3.3

Let *H* be a weakly norming graph without isolated vertices and let *F* be its subgraph. Then ${e(F)}/{v(F)}\le {e(H)}/{v(H)}$.

### Proof

We may assume *F* has no isolated vertices either, as adding isolated vertices only reduces the average degree. Let $X\subseteq \Omega$ be a subset with $\nu (X)=1/2$ and let $U:\Omega ^2\rightarrow [0,1]$ be the graphon defined by $U(x,y)=1$ if $x,y\in X$ and 0 otherwise. Then, for any graph *J* without isolated vertices, $t(J,U)=2^{-v(J)}$. Choosing $W_e=U$ for $e\in E(F)$ and $W_e\equiv 1$ otherwise, for ${\mathbf {w}}\in {\mathscr {W}}_{\ge 0}^{E(H)}$ then gives

$$\begin{aligned} t(F,U)^{e(H)}=t(H,{\mathbf {w}})^{e(H)}\le t(H,U)^{e(F)}t(H,1)^{e(H)-e(F)}=t(H,U)^{e(F)}. \end{aligned}$$

Comparing $t(F,U)^{e(H)}=2^{-v(F)e(H)}$ and $t(H,U)^{e(F)}=2^{-v(H)e(F)}$ concludes the proof. $\square$

### Remark 3.4

This is reminiscent of [5, Thm. 2.10(i)]. It states that ${e(F)}/({v(F)-1})\le {e(H)}/({v(H)-1})$ whenever *H* is weakly norming and *F* is a subgraph of *H* with $v(F)>1$. However, this theorem is only true if *H* is connected and hence also needs to be corrected. To see this, let *H* be a vertex-disjoint union of two copies of $K_{1,2}$, which is a norming graph. Then ${e(H)}/({v(H)-1})=4/5$ but ${e(F)}/({v(F)-1})=1$ for $F=K_{1,2}$.

Suppose now that a weakly norming graph *H* without isolated vertices consists of two vertex-disjoint subgraphs $F_1$ and $F_2$. If $e(F_1)/v(F_1)>e(F_2)/v(F_2)$, then

$$\begin{aligned} \frac{e(H)}{v(H)} = \frac{e(F_1)+e(F_2)}{v(F_1)+v(F_2)}<\frac{e(F_1)}{v(F_1)}, \end{aligned}$$

which contradicts Lemma 3.3. By iterating this, we obtain the following fact.

### Corollary 3.5

Every component in a weakly norming graph without isolated vertices has the same average degree.

Before proceeding to the next step, we recall some basic facts about $\ell ^p$-spaces. For $0<p<q\le +\infty$ we have $\Vert \,{\cdot }\,\Vert _p \ge \Vert \,{\cdot }\,\Vert _q$. Furthermore, there exists ${\mathbf {c}}\in \ell ^\infty$ such that

3.2 $$\begin{aligned} \Vert {\mathbf {c}} \Vert _p > \Vert {\mathbf {c}} \Vert _q. \end{aligned}$$

### Lemma 3.6

In a weakly norming graph *H* without isolated vertices, every connected component has the same number of edges.

### Proof

Let $F_1,\ldots ,F_k$ be the connected components of *H* and let $\gamma \,{:}{=}\,{v(F_1)}/{e(F_1)}$. By Corollary 3.5, $2/{\gamma }$ is the average degree of all $F_i$, $i=1,\ldots ,k$. Recall the definition of $W_{\gamma ,{\mathbf {a}}}$ given in Definition 2.4. For each ${\mathbf {a}}=(a_1,a_2,\ldots )\in \ell ^\infty$ and each connected graph *F* also having average degree $2/{\gamma }$, and, say, *m* edges, we have

3.3 $$\begin{aligned} t(F,|W_{\gamma ,{\mathbf {a}}}|)=\sum _i |a_i|^{m}=\Vert {\mathbf {a}} \Vert _{m}^{m}. \end{aligned}$$

Suppose that not all the components have the same number of edges. Let $p=\min _j e(F_j)$. We may assume that $p=e(F_1)$. Let $q>p$ be the number of edges in a component with more edges than $F_1$ and let ${\mathbf {c}}\in \ell ^\infty$ be given by (3.2). Define the collection ${\mathbf {w}}=(W_e)_{e\in E(H)}$ by $W_e=|W_{\gamma ,{\mathbf {c}}}|$ for $e\in E(F_1)$ and $W_e\equiv 1$ otherwise. Lemma 3.2 then gives

3.4 $$\begin{aligned} t(F_1,|W_{\gamma ,{\mathbf {c}}}|)^{e(H)}=t(H,{\mathbf {w}})^{e(H)}\,\le \! \prod _{e\in E(H)}\!t(H,W_e)=t(H,|W_{\gamma ,{\mathbf {c}}}|)^{p}. \end{aligned}$$

Expanding the term $t(H,|W_{\gamma ,{\mathbf {c}}}|)$ on the right-hand side of (3.4) using (3.3) yields

$$\begin{aligned} t(H,|W_{\gamma ,{\mathbf {c}}}|)=\prod _{j=1}^k t(F_j,|W_{\gamma ,{\mathbf {c}}}|)=\prod _{j=1}^k \Vert {\mathbf {c}}\Vert _{e(F_j)}^{e(F_j)}. \end{aligned}$$

On the left-hand side of (3.4), we have by (3.3) that $t(F_1,|W_{\gamma ,{\mathbf {c}}}|)=\Vert {\mathbf {c}}\Vert _{p}^{p}$. Substituting these back to (3.4) gives

$$\begin{aligned} \Vert {\mathbf {c}}\Vert _{p}^{p\cdot e(H)}\le \left( \,\,\prod _{j=1}^k \Vert {\mathbf {c}}\Vert _{e(F_j)}^{e(F_j)}\right) ^{\!p}, \end{aligned}$$

which contradicts the fact that $\Vert {\mathbf {c}}\Vert _{p}\ge \Vert {\mathbf {c}}\Vert _{e(F_j)}$ for each $j\in [k]$ with at least one of the inequalities being strict. $\square$

### Lemma 3.7

For a weakly norming graph *H* without isolated vertices, all the components of *H* are isomorphic.

### Proof

Suppose that there are at least two non-isomorphic graphs amongst all the components $F_1,\ldots ,F_k$. By Lemma 3.6 we may assume that all $F_i$ have the same number of edges, say *m*. In particular, $e(H)=mk$. By [8, Thm. 5.29], there exists a graphon *U* such that the numbers $t(F_1,U),\ldots ,t(F_k,U)$ are not all equal. We may assume that $t(F_1,U)$ attains the maximum amongst $t(F_1,U),\ldots ,t(F_k,U)$. Then we have $t(H,U)=\prod _{i=1}^k t(F_i,U)<t(F_1,U)^k$, in contradiction with

$$\begin{aligned} t(F_1,U)\le t(H,U)^{m/e(H)}= t(H,U)^{1/k}, \end{aligned}$$

which follows from (3.1). $\square$

### Proof of Theorem 1.2

Suppose first that *H* is weakly norming. Let *F* be the graph given by Lemma 3.7, which is isomorphic to every component of *H*, and let *k* be the number of components of *H*. Now enumerate the edges in *H* by $(e,i)\in E(F)\times [k]$, where each (*e*, *i*) denotes the edge *e* in the *i*-th copy of *F*. Then each ${\mathbf {w}}\in {\mathscr {W}}^{E(H)}$ can be written as $({\mathbf {w}}_1,\ldots ,{\mathbf {w}}_k)$, where ${\mathbf {w}}_i = (W_{e,i})_{e\in E(F)}$, so that $t(H,{\mathbf {w}})=\prod _{i=1}^kt(F,{\mathbf {w}}_i)$. Let ${\mathbf {u}}=(U_e)_{e\in E(F)}\in {\mathscr {W}}_{\ge 0}^{E(F)}$ be arbitrary. Then Lemma 3.2 together with the choice ${\mathbf {w}}_1=\cdots ={\mathbf {w}}_k={\mathbf {u}}$, i.e., $W_{e,i}=U_e$, implies

3.5 $$\begin{aligned} t(F,{\mathbf {u}})^{k^2\cdot e(F)}&=t(F,{\mathbf {u}})^{k\cdot e(H)}=t(H,{\mathbf {w}})^{e(H)}\\&\le \!\prod _{(e,i)\in E(H)}\!\!t(H,W_{e,i})\,=\!\prod _{(e,i)\in E(F)\times [k]}\!\!\!t(F,U_{e})^k\,=\!\prod _{e\in E(F)}\!t(F,U_{e})^{k^2}.\nonumber \end{aligned}$$

Taking the $k^2$-th root proves that *F* is weakly norming.

When *H* is semi-norming, we can still apply Lemma 3.7 to obtain a graph *F* isomorphic to each component, since *H* is also weakly norming. Thus, the enumeration $E(F)\times [k]$ of *E*(*H*) and the factorisation $t(H,{\mathbf {w}})=\prod _{i=1}^k t(F,{\mathbf {w}}_i)$ for each ${\mathbf {w}}=({\mathbf {w}}_1,\ldots ,{\mathbf {w}}_k)\in {\mathscr {W}}^{E(H)}$ remain the same. Now let ${\mathbf {u}}=(U_f)_{f\in E(F)}\in {\mathscr {W}}^{E(F)}$ be arbitrary. Then, again by taking ${\mathbf {w}}_1=\cdots ={\mathbf {w}}_k={\mathbf {u}}$ in Lemma 3.2, we obtain

$$\begin{aligned} t(F,{\mathbf {u}})^{k^2\cdot e(F)}=t(H,{\mathbf {w}})^{e(H)}\,\le \!\prod _{(e,i)\in E(H)}\!|t(H,W_{e,i})|\,=\!\prod _{e\in E(F)}\!|t(F,U_{e})|^{k^2}, \end{aligned}$$

which proves that *F* is semi-norming. If *H* is norming, then $|t(F,W)|=|t(H,W)|^{1/k}$ must be nonzero for each nonzero $W\in {\mathscr {W}}$. Thus, *F* is also norming. $\square$

## Concluding Remarks

As mentioned in Sect. 2, Theorem 1.2 yields a full generalisation of Theorem 2.2.

### Theorem 4.1

For each $m\in {\mathbb {N}}$, there exist constants $C_m,C'_m>0$ such that the following holds: let *H* be a semi-norming graph with *m* edges in each (isomorphic) non-singleton component. Then the Banach space $({\mathscr {W}}_H,\Vert \,{\cdot }\,\Vert _H)$ satisfies $C_m\cdot {\mathfrak {d}}_{\ell ^m}\le {\mathfrak {d}}_{H}\le {\mathfrak {d}}_{\ell ^m}$ and ${\mathfrak {s}}_{\ell ^m}\le {\mathfrak {s}}_{H}\le C'_m\cdot {\mathfrak {s}}_{\ell ^m}$.

As a consequence, the connectivity condition in Corollary 2.3 can also be removed, i.e., $({\mathscr {W}}_H,\Vert \,{\cdot }\,\Vert _H)$ is always uniformly convex and uniformly smooth whenever *H* is semi-norming.

There is more literature in the area that has been imprecise when it comes to connectivity, but which can be corrected with Theorem 1.2 to hold in full generality. For instance, [8, Exercise 14.7 (b)] states that every semi-norming graph is either a star or an Eulerian graph, which is true only if the semi-norming graph is connected. To correct the statement, we may replace a star by a vertex-disjoint union of isomorphic stars by using Theorem 1.2. Likewise, whenever studying properties of graph norms, one can invoke Theorem 1.2 and focus on connected graphs. We finally remark that the theorems used in our proofs have no errors concerning connectivity. In particular, [5, Thm. 2.8] is still valid regardless of connectivity.

In [6], the *step Sidorenko property* is defined to prove that there exists an edge-transitive graph that is not weakly norming (for the precise definition we refer to [6]), where the proof relies on the fact that every weakly norming graph is step Sidorenko (see [8]). Moreover, it is shown in [4] that the converse is also true for connected graphs, i.e., every connected step Sidorenko graph is weakly norming. However, Theorem 1.2 proves that the converse no longer holds for disconnected graphs, as a vertex-disjoint union of non-isomorphic step Sidorenko graphs is again step Sidorenko but not weakly norming.

## Footnotes

1 By the topological equivalence between the cut norm and graph norms (see, for instance, [2, Sect. 5.2]) and completeness of ${\mathscr {W}}$ under the cut norm, $\Vert \,{\cdot }\,\Vert _{r(H)}$ and $\Vert \,{\cdot }\,\Vert _H$ also define Banach spaces.
